# Quantitative Optical Coherence Tomography Angiography Detects Retinal Perfusion Changes in Carotid Artery Stenosis

**DOI:** 10.3389/fnins.2021.640666

**Published:** 2021-04-22

**Authors:** Luisa Pierro, Alessandro Arrigo, Michele De Crescenzo, Emanuela Aragona, Roberto Chiesa, Renata Castellano, Barbara Catenaccio, Francesco Bandello

**Affiliations:** ^1^Department of Ophthalmology, Scientific Institute San Raffaele Hospital, University Vita-Salute, Milan, Italy; ^2^Department of Surgery, San Raffaele Hospital, Milan, Italy

**Keywords:** OCT, OCTA, Retina, Endarterectomy, Carotid artery stenosis (CAS)

## Abstract

**Background:**

Carotid artery stenosis (CAS) is a multifaceted disease characterized by possible ocular involvement. Treatment with carotid endarterectomy helps to restore cerebral perfusion, which may prevent ocular and cerebral complications. The main aim was to assess retinal and choroidal vascular perfusion changes before and after endarterectomy in patients affected by CAS.

**Methods:**

The design of the study was prospective and observational, including patients affected by CAS and healthy controls. The follow-up was 3 months. We performed quantitative optical coherence tomography (OCT) angiography (OCTA) analyses of retinal perfusion changes, before and after endarterectomy. The main outcome measures were the quantitative changes of choroidal thickness (CT), retinal nerve fiber layer (RNFL), and ganglion cell layer (GCL); vessel density (VD); and vessel tortuosity (VT) OCTA metrics were also measured.

**Results:**

Sixty eyes of 30 patients affected by CAS and 30 eyes of 30 controls were included. We separately considered the ipsilateral eyes to CAS, the contralateral eyes to CAS, and the healthy eyes. Visual symptoms were absent in all the patients. RNFL and GCL resulted similar between patients and controls (*p* > 0.05). CT was significantly thinner in ipsilateral eyes than controls (*p* < 0.01), and it resulted unchanged after surgery (*p* > 0.05). VD resulted significantly altered only in some plexa of the ipsilateral eyes (*p* < 0.01), whereas VT disclosed decreased values of the entire retinal vascular network, both in ipsilateral and contralateral eyes (*p* < 0.05). Endarterectomy was followed by statistically significant improvement of retinal perfusion (*p* < 0.05).

**Conclusion:**

Optical coherence tomography angiography can noninvasively detect postendarterectomy retinal perfusion improvements in CAS patients with baseline diabetes and hypertension as a systemic risk factor.

## Introduction

Carotid artery stenosis (CAS) is a complex disease that can lead to a broad spectrum of neurological disorders such as ischemic stroke, transient ischemic attack, and vascular dementia (Grotta, 2013). As the ophthalmic artery directly arises from the internal carotid artery, ophthalmic manifestations can be commonly found in CAS (Cohen et al., 2010). Amaurosis fugax, permanent occlusions, venous stasis retinopathy, and ocular ischemic syndrome might lead to severe visual impairment in patients affected by CAS (Cohen et al., 2010).

Several imaging tests have been employed in the past years to evaluate the ocular changes associated with CAS. Color Doppler imaging can be used to detect reduction of blood flow in the ophthalmic artery and its improvement after surgical treatment (Mawn et al., 1997; Costa et al., 1999; Kawaguchi et al., 2002). Fluorescein angiography has been reported as a useful investigation to detect microvascular changes associated with CAS, although the invasive nature discourages its usage in patients without ocular symptoms (Choromokos et al., 1982; Sarkies et al., 1987; Terelak-Borys et al., 2012).

Optical coherence tomography angiography (OCTA) is a recent imaging technique that can rapidly and noninvasively provide angiograms of the intraretinal vascular network. OCTA images provide accurate information regarding the perfusion status of intraretinal capillaries, and it is extremely sensitive in detecting retinal vascular alteration (Arrigo et al., 2018; Battaglia Parodi et al., 2018; Parodi et al., 2018; Pierro et al., 2018, 2019a, Pierro et al., 2019b; Spaide et al., 2018; Pierro et al., 2020a). The introduction of quantitative OCTA approaches is extremely useful in improving the detection of optic nerve head vascular network changes (Pierro et al., 2020b). To date, the retinal assessment in CAS setting was mainly confined to conventional angiography, which is invasive and prone to possible side effects. On the contrary, OCTA is a feasible and noninvasive approach to detect and quantify retinal perfusion changes. However, few data are available in the literature regarding the use of OCTA in CAS clinical setting. Furthermore, the recent development of new quantitative OCTA biomarkers provided a step forward in the assessment of retinal perfusion features, including vessel tortuosity (VT); this can be considered an indirect measure of the amount of blood flow interesting intraretinal capillaries (Arrigo et al., 2018, 2020). To the best of our knowledge, this parameter was never employed in a surgical setting such as CAS endarterectomy.

The purpose of this study was to employ quantitative OCTA to analyze retinal perfusion changes in patients affected by CAS before and after endarterectomy surgical intervention.

## Materials and Methods

Thirty patients (60 eyes; 17 males; mean age 68 ± 8 years) affected by CAS were recruited in the period from January 2018 to September 2019. Both ipsilateral and contralateral eyes to CAS were separately considered for the quantitative analyses. Moreover, 30 healthy controls (30 eyes; 16 males; mean age 65 ± 10 years) were recruited. Each patient provided signed informed consent before the examination. The study was conducted in accordance with the Declaration of Helsinki, and it was approved by the ethical committee of the Vita-Salute San Raffaele University of Milan (MIRD2020). The diagnosis and degree of CAS were previously detected using eco-color Doppler (ECST criteria). The patients were classified according to the percentage of internal CAS and the morphological characteristics of the plaque.

Inclusion criteria were as follows: ultrasound confirmation of CAS; surgical treatment planning, according to The Society for Vascular Surgery’s guidelines (Ricotta et al., 2011) (symptomatic patients with stenosis of 50–99%; asymptomatic patients with stenosis of 60–99%; <3% perioperative risk of stroke and death in asymptomatic patients; life expectancy of 3–5 years).

Exclusion criterion was the identification of any condition that could affect the analyses: refractive status > ±3D, media opacities, age-related macular degeneration, retinal vascular diseases, retinal dystrophies, previous ocular surgery in the last 6 months, or any systemic condition potentially affecting the findings such as uncontrolled diabetes mellitus and arterial hypertension. Those conditions were ruled out also in the control group.

All the patients underwent complete ophthalmological evaluation including best-corrected visual acuity (BCVA) measurement, biomicroscopic examination, intraocular pressure measurement by Goldmann applanation tonometry, and slit-lamp fundus examination. Structural OCT and OCTA scans were performed by means of a DRI OCT Topcon Triton (Topcon Corp., Japan). Only high-quality images were included, assessed by Topcon quality index > 80, included into device software. Poor-quality acquisitions were eventually repeated. For the quantitative analysis, we considered only the best-quality acquisition, showing the highest Topcon quality index. The acquisitions were performed before surgery (baseline), the day after surgery (after treatment), and 3 months after surgery (last follow-up). All 30 patients completed all three sets of data collection.

Structural OCT scans included radial and raster images. 3D wide (H) (12 × 9 mm) scans were also acquired to obtain average thicknesses of the RNFL and the GCL. CT was measured using horizontal structural OCT scan centered on the fovea. Measurements of the fovea were taken under the center of the fovea, at 750 μm, and at 1,500 μm (nasal and temporal sides), for a total of five measures. The mean measure was included into the statistical analyses.

OCTA scans included 4.5 × 4.5 and 9 × 9-mm scans centered on the fovea and 4.5 × 4.5-mm OCTA scans centered on the optic nerve head; the angiograms from superficial (SCP), deep capillary (DCP), choriocapillaris (CC), and radial peripapillary capillaries (RPC) plexuses were automatically segmented by ImageNET software and eventually manually corrected by an expert ophthalmologist (A.A.). The automatic segmentations resulted accurate in 100% of cases and required no further changes. To improve readability, we marked macular plexa with “m” and optic nerve head ones with “n.”

The segmented images were exported and loaded to ImageJ 1.50 software (National Institutes of Health, Bethesda, MD, United States). In-house scripts were built to calculate VD and VT. The “adjust threshold” tool was used in ImageJ to highlight the blood vessels and to reduce the noise. VD was calculated after image binarization, obtained by applying a “mean” threshold for each reconstruction and then by calculating the percentage of white pixels against black and blue ones, the latter resulting from the segmentation of the foveal avascular zone, conventionally considered as exclusion criterion. This procedure was adopted for all the eyes.

Vessel tortuosity was calculated after applying the “skeletonize” function to all reconstructions, enabling each vessel to be considered as a line. Then, we applied the ImageJ pipeline named “Analyze skeleton” to extract VT values.

The surgery was performed in the Department of Vascular Surgery (San Raffaele Hospital; Milan, Italy), carried out under cervical plexus anesthesia. After systemic heparinization, the common, the internal, and the external carotid arteries were isolated and prepared. Clamping of the internal, common, and external carotid artery was then performed, followed by arteriotomy and section of the internal carotid artery. Eversion endarterectomy of the internal, common, and external carotid artery was then performed. Afterward, reimplantation of the internal carotid artery on the common carotid artery by continuous suture was carried out followed by sequential declamping of the external, common, and internal carotid arteries. After an angiographic check, an accurate hemostasis was performed, a drainage was placed, and cervicotomy was sutured. Patients were then monitored for at least 24 h.

Two expert blinded graders (A.A., E.A.) performed all the measures. Interclass correlation coefficient (ICC) was tested by means of a two-way mixed model. OCTA acquisitions were performed at least twice to test both repeatability and reproducibility of the quantitative metrics.

All the data were analyzed using SPSS version 23 (SPSS Inc., Chicago, IL, United States). For the statistical analysis, age and gender were considered as fixed factors. We evaluated the normality distribution of each variable with quantile–quantile plots and frequency histograms. Shapiro–Wilk test showed that all the variables were included within the 95% of the confidence interval. Descriptive statistics of continuous variables were reported as mean ± standard deviation. Systolic and diastolic blood pressure preoperative values were considered as covariate for the analyses. Although belonging to the same patients, the eyes on the same side of CAS and the contralateral eyes were separately measured, to assess the effect of endarterectomy separately for both subgroups of eyes. Continuous variables were analyzed by means of a two-tailed *t* test (mean values of CAS eyes vs. healthy controls). Tau–Kendall correlation analysis was adopted to assess the statistical relationship among all the considered variables. The Bonferroni approach was employed to address the question of multiple comparisons. The statistical significance threshold was set to *p* ≤ 0.05.

## Results

**FIGURE 1:**
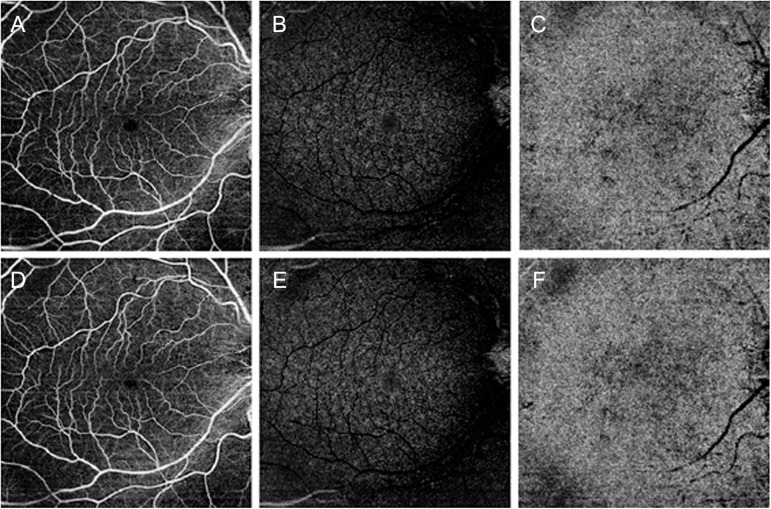
Optical coherence tomography angiography (OCTA) images of a patient affected by carotid artery stenosis (CAS). Before surgery, OCTA qualitatively disclosed unremarkable changes for superficial capillary plexus in panel **(A)**, deep capillary plexus in panel **(B)**, and choriocapillaris in panel **(C)**. After endarterectomy, qualitative OCTA was comparable to presurgical images for all the three plexa **(D–F)**, respectively.

All the 30 patients were affected by unilateral CAS. Mean BCVA was 0.0 ± 0.0 logMAR for both ipsilateral and contralateral eyes, as well as for the control group (*p* > 0.05). Demographic and clinical data are reported in Table 1. Systolic blood pressure was 140 ± 20 mm Hg, whereas diastolic blood pressure was 76 ± 14 mm Hg, under pharmacological treatment. The qualitative evaluation of OCTA images did not macroscopically reveal retinal vascular alterations associated with CAS (Figure 1). Five patients complained ischemic symptoms not involving the eye; the ischemic event occurred on average 43 ± 12 days prior to the baseline examination. No patient showed signs of postoperative ischemia.

**TABLE 1:** Demographic and clinical data.

Demographic and clinical data				
	Ipsilateral eye (1)	Contralateral eye (2)	Control eye (3)	*p* value
Age	68 ± 8		65 ± 10	>0.05
Gender (male/female)	17/13		16/14	>0.05
LogMAR BCVA	0.0 ± 0.0	0.0 ± 0.0	0.0 ± 0.0	>0.05
Arterial hypertension	30/30 (100%)		0/30 (0%)	<0.05*
Diabetes mellitus	12/30 (40%)		0/30 (0%)	<0.05*
Previous cerebral vascular accident	0/30 (0%)		0/30 (0%)	>0.05
Previous transitory ischemic attack	5/30 (17%)		0/30 (0%)	<0.05*

Statistically significant values are marked by asterisks (*).

Repeatability and reproducibility for the performed measures were overall 0.89 (range 0.87–0.94) and 0.92 (range 0.89–0.95), respectively. Overall ICC was 0.91 (range 0.88–0.95; *p* < 0.01).

Choroidal thickness was found significantly thinner in the ipsilateral eyes at baseline, with respect to controls (223.321 ± 67.37 vs. 319.28 ± 60.76 μm; *p* < 0.01) and disclosed unchanged values after surgery (*p* > 0.05) (Table 2). The CT of the contralateral eyes was always similar to controls (*p* > 0.05).

**TABLE 2:** Structural optical coherence tomography (OCT) analysis in carotid artery stenosis (CAS).

Structural OCT analysis			
	Ipsilateral eye (1)	Contralateral eye (2)	Control eye (3)
CT baseline	223.321 ± 67.37	285.42 ± 65.65	319.28 ± 60.76
CT final	224.443 ± 68.83	302.23 ± 66.98	
RNFL thickness	102.64 ± 10.25	105.45 ± 10.42	107.32 ± 9.86
GCL thickness	68.59 ± 7.69	70.55 ± 6.02	72.34 ± 5.56

***p* values**	**1 vs. 2**	**1 vs. 3**	**2 vs. 3**

CT baseline	<0.05*	<0.05*	>0.05
CT final	<0.05*	<0.05*	>0.05
RNFL thickness	>0.05	>0.05	>0.05
GCL thickness	>0.05	>0.05	>0.05

RNFL, retinal nerve fiber layer; GCL, ganglion cell layers; CT, choroidal thickness. Statistically significant values are marked by asterisks (*).

Retinal nerve fiber layer thickness was found to be 103 ± 10 μm for the ipsilateral eye, 105 ± 10 μm for the contralateral eye and 107 ± 10 μm for the control group (*p* > 0.05), and it resulted unchanged after surgery (*p* > 0.05) (Table 2).

Ganglion cell layer thickness resulted 69 ± 8 μm for the ipsilateral eye, 71 ± 6 μm for the contralateral eye, and 72 ± 6 for the control group (*p* > 0.05), and also in this case, it resulted unchanged after surgery (*p* > 0.05) (Table 2).

Vessel density analysis disclosed unremarkable differences among the three groups for SCP and CC (*p* > 0.05) (Table 3). With respect to DCP and RPC, both macular and optic nerve head plexa disclosed significantly lower VD values in the ipsilateral eye at baseline, with respect to both contralateral eyes and control eyes (*p* < 0.01). After surgery, both plexa disclosed a significant improvement, turning out to be statistically comparable with the other two groups (*p* > 0.05) (Table 3).

**TABLE 3:** Vessel density OCTA analysis in CAS.

Vessel density analysis							
		Ipsilateral eye (1)	Contralateral eye (2)	Control eye (3)	*p* values		
					1 vs. 2	1 vs. 3	2 vs. 3
VD mSCP	Baseline	0.41 ± 0.01	0.41 ± 0.01	0.41 ± 0.01	>0.05	>0.05	>0.05
	Post treatment	0.41 ± 0.02	0.41 ± 0.02		>0.05	>0.05	>0.05
	Last follow-up	0.41 ± 0.01	0.41 ± 0.01		>0.05	>0.05	>0.05
	*p* value	>0.05	>0.05				
VD mDCP	Baseline	0.41 ± 0.02	0.42 ± 0.02	0.43 ± 0.01	<0.05*	<0.05*	>0.05
	Post treatment	0.43 ± 0.03	0.43 ± 0.02		>0.05	>0.05	>0.05
	Last follow-up	0.43 ± 0.02	0.43 ± 0.02		>0.05	>0.05	>0.05
	*p* value	<0.05*	>0.05				
VD mCC	Baseline	0.50 ± 0.02	0.50 ± 0.02	0.50 ± 0.02	>0.05	>0.05	>0.05
	Post treatment	0.50 ± 0.03	0.50 ± 0.02		>0.05	>0.05	>0.05
	Last follow-up	0.50 ± 0.02	0.50 ± 0.02		>0.05	>0.05	>0.05
	*p* value	>0.05	>0.05				
VD RPC	Baseline	0.42 ± 0.02	0.43 ± 0.02	0.45 ± 0.01	<0.05*	<0.05*	>0.05
	Post treatment	0.44 ± 0.02	0.44 ± 0.02		>0.05	>0.05	>0.05
	Last follow-up	0.44 ± 0.02	0.44 ± 0.02		>0.05	>0.05	>0.05
	*p* value	<0.05*	>0.05				
VD nSCP	Baseline	0.43 ± 0.02	0.430.02	0.43 ± 0.01	>0.05	>0.05	>0.05
	Post treatment	0.43 ± 0.02	0.43 ± 0.02		>0.05	>0.05	>0.05
	Last follow-up	0.43 ± 0.02	0.43 ± 0.02		>0.05	>0.05	0.05
	*p* value	>0.05	>0.05				
VD nDCP	Baseline	0.37 ± 0.02	0.38 ± 0.02	0.39 ± 0.01	<0.05*	<0.05*	>0.05
	Post treatment	0.39 ± 0.02	0.39 ± 0.02		>0.05	>0.05	>0.05
	Last follow-up	0.39 ± 0.02	0.39 ± 0.02		>0.05	>0.05	>0.05
	*p* value	<0.05*	>0.05				
VD nCC	Baseline	0.54 ± 0.02	0.54 ± 0.02	0.54 ± 0.02	>0.05	>0.05	>0.05
	Post treatment	0.54 ± 0.02	0.54 ± 0.02		>0.05	>0.05	>0.05
	Last follow-up	0.54 ± 0.02	0.54 ± 0.02		>0.05	>0.05	>0.05
	*p* value	>0.05	>0.05				

VD, vessel density; SCP, superficial capillary plexus; DCP, deep capillary plexus; CC, choriocapillaris; RPC, radial peripapillary capillaries plexus. “m” and “n” refer to macular and optic nerve head plexa, respectively. Statistically significant values are marked by asterisks (*).

It is worth of notice that VT values resulted significantly lower both in ipsilateral and contralateral eyes, with respect to controls (*p* < 0.05) (Table 4).

**TABLE 4:** Vessel tortuosity OCTA analysis in CAS.

Vessel tortuosity analysis							
		Ipsilateral eye (1)	Contralateral eye (2)	Control eye (3)	*p* values		
					1 vs. 2	1 vs. 3	2 vs. 3
VT mSCP	Baseline	6.86 ± 0.5	7.02 ± 0.4	7.18 ± 0.03	<0.05*	<0.05*	<0.05*
	Post treatment	7.19 ± 0.5	7.19 ± 0.3	7.18 ± 0.03	>0.05	>0.05	>0.05
	Last follow-up	7.19 ± 0.5	7.19 ± 0.3	7.18 ± 0.03	>0.05	>0.05	>0.05
	*p* value	<0.05*	<0.05*	N/A			
VT mDCP	Baseline	7.48 ± 0.6	7.66 ± 0.5	7.83 ± 0.03	<0.05*	<0.05*	<0.05*
	Post treatment	7.85 ± 0.4	7.84 ± 0.3	7.83 ± 0.03	>0.05	>0.05	>0.05
	Last follow-up	7.84 ± 0.4	7.83 ± 0.3	7.83 ± 0.03	>0.05	>0.05	>0.05
	*p* value	<0.05*	<0.05*	N/A			
VT RPC	Baseline	7.37 ± 0.5	7.54 ± 0.3	7.71 ± 0.03	<0.05*	<0.05*	<0.05*
	Post treatment	7.73 ± 0.4	7.73 ± 0.3	7.71 ± 0.03	>0.05	>0.05	>0.05
	Last follow-up	7.73 ± 0.4	7.73 ± 0.3	7.71 ± 0.03	>0.05	>0.05	>0.05
	*p* value	<0.05*	<0.05*	N/A			
VT nSCP	Baseline	8.04 ± 0.4	8.23 ± 0.4	8.42 ± 0.2	<0.05*	<0.05*	<0.05*
	Post treatment	8.42 ± 0.4	8.43 ± 0.3	8.42 ± 0.2	>0.05	>0.05	>0.05
	Last follow-up	8.43 ± 0.4	8.43 ± 0.3	8.42 ± 0.2	>0.05	>0.05	>0.05
	*p* value	<0.05*	<0.05*	N/A			
VT nDCP	Baseline	6.72 ± 0.5	6.88 ± 0.4	7.04 ± 0.3	<0.05*	<0.05*	<0.05*
	Posttreatment	7.06 ± 0.4	7.04 ± 0.3	7.04 ± 0.3	>0.05	>0.05	>0.05
	Last follow-up	7.05 ± 0.4	7.05 ± 0.3	7.04 ± 0.3	>0.05	>0.05	>0.05
	*p* value	<0.05*	<0.05*	N/A			

VT, vessel tortuosity; SCP, superficial capillary plexus; DCP, deep capillary plexus; CC, choriocapillaris; RPC, radial peripapillary capillaries plexus. “m” and “n” refer to macular and optic nerve head plexa, respectively. Statistically significant values are marked by asterisks (*).

Ipsilateral eyes disclosed the worst VT values (*p* < 0.01) (Table 4). Both ipsilateral and contralateral eyes showed a statistically significant VT improvement after surgery (*p* < 0.05), maintaining significantly lower values than control eyes (*p* < 0.05). Furthermore, also at the end of the follow-up, ipsilateral eyes disclosed the lowest VT values (*p* < 0.05) (Table 4). We did not find any significant correlation between the degree of CAS and the OCTA parameters (all *p* > 0.05) (data not shown).

## Discussion

Carotid artery stenosis can cause a decrease in ophthalmic artery flow with the onset of ocular complication (Cohen et al., 2010). The presence of anastomoses network of the ophthalmic artery makes ocular involvement an uncommon phenomenon; however, it is crucial to identify patients at risk because of the potential irreversible nature of retinal damage (Cohen et al., 2010).

In the present study, we showed how quantitative OCTA analyses could detect retinal perfusion changes already in asymptomatic eyes. Remarkably, the qualitative assessment showed no evident changes of the intraretinal vascular network. On the other side, the employment of quantitative parameters, namely, VD and VT, allowed to detect statistically significant changes related to retinal blood flow perfusion modifications, occurring before and after endarterectomy. Significant improvements of VT metrics are worth of notice, detected already after only 1 day since endarterectomy. This may be interpreted as a prompt positive effect of the treatment on the overall capillary perfusion, which turned out to be maintained also after 3 months. The normal VD values characterizing ipsilateral eyes can be justified by the fact that VD is a parameter focused on the presence or absence or retinal capillaries. For this reason, major changes are needed to determine significant variations of VD values. On the contrary, VT is more dedicated on the quantification of blood flow perfusion, which can influence the course of the capillaries, thus making these variably tortuous. VD resulted altered only in DCP and RPC, which are well-known to be earlier involved in several retinal diseases, because of the lower perfusion pressure and the extremely thin morphology (Arrigo et al., 2018). In particular, DCP is a low-pressure network, originating from the SCP and receiving blood from branches of the central retinal artery. On this basis, it is expected that a perfusion pressure reduction could primarily affect DCP. On the other side, RPC is made of thin capillaries running within the thickness of the retinal nerve fibers and resulting supply by branches of the ciliary arteries. Because of the peculiar anatomy, origin, and blood supply, RPC is recognized as a network earlier influenced by perfusion dropout (Mase et al., 2016).

On the other side, VT parameter can assess the presence of earlier perfusion alterations, on the basis of tortuosity values reflecting the amount of blood flow passing through the capillaries (Arrigo et al., 2018, 2020; Pierro et al., 2020b).

Our study provided also insights regarding the effect of CAS surgery on retinal perfusion. Indeed, it was possible to quantify the amount of perfusion recovery, secondary to endarterectomy and the presence of residual perfusion deficit. Interestingly, we also detected perfusion improvements also in the contralateral eye. This might be the consequence of an overall improvement of blood flow circulation secondary to the conventional pharmacological anticoagulant/antiaggregant therapy administered before and after surgery.

To the best of our knowledge, only one investigation has been conducted in order to assess OCTA changes in CAS patients (Lahme et al., 2018). This study agreed in terms of including only asymptomatic patients, although their follow-up was limited just to 3–4 days after surgery, whereas our follow-up was longer (Lahme et al., 2018). This previous investigation disclosed similar results regarding RPC blood flow impairment before surgery and its restoration after endarterectomy in the ipsilateral. On the other side, the authors described a significant perfusion involvement also of the contralateral eye, which resulted poorly detectable in our investigation.

Some contradictory results came also from structural OCT analyses. Our structural OCT investigation detected no remarkable changes of retinal layers. With respect to RNFL thickness, previous investigations lead to contradictory results. Two different studies reported that mean thicknesses of the RNFL, macula, and GCC did not differ between patients with CAS and age-matched healthy controls (Heßler et al., 2015; Sayin et al., 2015). On the contrary, other investigations reported significant macular and RNFL thinning in CAS patients, also in asymptomatic cases (Wang et al., 2017; Çakir et al., 2017).

Regarding CT, there is controversial evidence in the literature too, with reports disclosing significant choroidal thinning or preserved CT (Sayin et al., 2015; Rabina et al., 2018). In our study, CT resulted significantly lower in CAS eyes. The lack of agreement about the choroidal involvement in CAS might be explained considering the anastomotic organization of ocular vasculature, including the differentiation of posterior ciliary arteries in branches, making the central retinal artery and branches directly perfusing the choroid (short posterior ciliary arteries). From this point of view, the amount of CAS might influence the functionality of this complex network, inducing a blood flow pressure drop at the level of the short posterior ciliary arteries, thus causing choroidal thinning. Based on our findings, this effect can be seen noninvasively through OCT and OCTA techniques. However, further investigations should be focused on the careful assessment of the relationship between the amount of CAS and the variations of the blood flow pressure of the posterior ciliary arteries.

Considering all the findings provided by our study and previous investigations, there is an overall agreement about the early involvement of retinal blood flow perfusion, related with CAS, also in asymptomatic eyes. On the other hand, the contradictory results might be related with limited number of recruited patients and probably high heterogeneity of coexisting ocular or systemic comorbidities. From this point of view, further prospective studies involving higher number of patients are warranted to draw definite conclusions. However, our study provided two steps forward regarding this topic, as it disclosed longer follow-up than previous investigations, and it combined both structural OCT and OCTA investigations, employing recently developed quantitative approaches. For these reasons, we believe our research was able to provide useful insights about the role of quantitative OCTA in detecting early retinal vascular alterations, in the perspective of development of diagnostic tests useful to optimize the stratification of the risk of cerebral ischemic events, as well as to better plan treatment strategies and outcome evaluations (Wolintz, 2005). From this point of view, the noninvasive nature of OCTA examination makes it a feasible candidate to stratify the risk of complications in CAS patients.

With respect to the relationship between structural OCT and OCTA parameters, we did not find significant correlations between RNFL/GCL thicknesses and VD/VT OCTA parameters. This might be due to the relatively low number of eyes. On the other side, we cannot exclude that the amount of blood flow perfusing the eye before endarterectomy turned out to be still enough to preserve the structural integrity of RNFL and GCL, thus not inducing irreversible damage.

We are aware that our study labors under several limitations. The limited number of eyes and the short follow-up might affect the results of our investigation. From this point of view, further larger studies are warranted to draw more definite conclusions about the ocular involvement in CAS clinical setting. Moreover, we limited our analyses only on asymptomatic eyes. This choice was done to assess the effective sensitivity of quantitative OCTA in detecting retinal perfusion changes secondary to CAS, without the interferences provided by the inclusion of symptomatic eyes with evident retinal damage. From this point of view, future study should be conducted to assess the amount of retinal impairment after the onset of ophthalmic symptoms in CAS patients, and possible improvements disclosed after surgery. Furthermore, OCTA is known to be subjected to several imaging artifacts (Spaide et al., 2018). We tried to assess this limitation by including only high-quality images. In addition, we did not include biometric assessment to measure axial length. Although we included only eyes with a refractive status < ±3D, quantitative OCTA parameters would have resulted more accurate if adjusted for axial length. Given the baseline differences between groups in regard to the prevalence of arterial hypertension and diabetes mellitus, a significant limitation of this study is the potential influence of these in the quantitative OCTA data. Future better controlled studies will help assess the reliability of OCTA to detect perfusion changes CAS surgical treatment. In addition, another limitation of the study is the lack of postoperative systolic and diastolic blood pressure values.

In conclusion, our quantitative OCTA investigation disclosed significant retinal vascular involvement in asymptomatic CAS patients and a remarkable retinal vascular recovery after surgery. Despite the preliminary nature of the study, our findings pave the stone for future extensive investigations on CAS patients in order to provide quantitative cutoff values for the evaluation of the risk of ocular involvement and to develop clinically useful prognostic biomarkers in CAS.

## Data Availability Statement

The datasets presented in this article are not readily available because they have been anonymized. Requests to access the datasets should be directed to AA, alessandro.arrigo@hotmail.com.

## Ethics Statement

The studies involving human participants were reviewed and approved by Ethical committee of San Raffaele Hospital, Milan. The patients/participants provided their written informed consent to participate in this study.

## Author Contributions

LP and AA: study the design, data acquisitions, data analysis, data interpretation, and manuscript draft. MD and EA: data analysis and data acquisition. RCa and BC: patients’ management and providing data. FB and RCh: data interpretation, manuscript revision, and study supervision. All authors contributed to the article and approved the submitted version.

## Conflict of Interest

FB is a consultant for: Alcon (Fort Worth, TX, United States), Alimera Sciences (Alpharetta, GA, United States), Allergan Inc (Irvine, CA, United States), Farmila-Thea (Clermont-Ferrand, France), Bayer Shering-Pharma (Berlin, Germany), Bausch and Lomb (Rochester, NY, United States), Genentech (San Francisco, CA, United States), Hoffmann-La-Roche (Basel, Switzerland), NovagaliPharma (Évry, France), Novartis (Basel, Switzerland), Sanofi-Aventis (Paris, France), Thrombogenics (Heverlee, Belgium), Zeiss (Dublin, OH, United States). The remaining authors declare that the research was conducted in the absence of any commercial or financial relationships that could be construed as a potential conflict of interest. The reviewer EM declared a past co-authorship with one of the authors AA and EA to the handling editor.
